# Identification of Toxic Pyrrolizidine Alkaloids and Their Common Hepatotoxicity Mechanism

**DOI:** 10.3390/ijms17030318

**Published:** 2016-03-07

**Authors:** Xinmiao Yan, Hong Kang, Jun Feng, Yiyan Yang, Kailin Tang, Ruixin Zhu, Li Yang, Zhengtao Wang, Zhiwei Cao

**Affiliations:** 1 School of Life Sciences and Technology, Tongji University, Shanghai 200092, China; 1434316@tongji.edu.cn (X.Y.); qilefeng525@163.com (J.F.); 1410775@tongji.edu.cn (Y.Y.); kltang@tongji.edu.cn (K.T.); rxzhu@tongji.edu.cn (R.Z.); 2 School of Biomedical Informatics, University of Texas Health Science Center, Houston, TX 77030, USA; kangh0607@gmail.com; 3 The Ministry of Education (MOE) Key Laboratory for Standardization of Chinese Medicines, Institute of Chinese Materia Medica, Shanghai University of Traditional Chinese Medicine, Shanghai 201203, China; yl7@shutcm.edu.cn (L.Y.); ztwang@shutcm.edu.cn (Z.W.)

**Keywords:** Pyrrolizidine Alkaloids, hepatotoxicity mechanism, glutathion metabolism, reverse docking

## Abstract

Pyrrolizidine Alkaloids (PAs) are currently one of the most important botanical hepatotoxic ingredients. Glutathion (GSH) metabolism is the most reported pathway involved in hepatotoxicity mechanism of PAs. We speculate that, for different PAs, there should be a common mechanism underlying their hepatotoxicity in GSH metabolism. Computational methods were adopted to test our hypothesis in consideration of the limitations of current experimental approaches. Firstly, the potential targets of 22 PAs (from three major PA types) in GSH metabolism were identified by reverse docking; Secondly, glutathione *S*-transferase A1 (GSTA1) and glutathione peroxidase 1 (GPX1) targets pattern was found to be a special characteristic of toxic PAs with stepwise multiple linear regressions; Furthermore, the molecular mechanism underlying the interactions within toxic PAs and these two targets was demonstrated with the ligand-protein interaction analysis; Finally, GSTA1 and GPX1 were proved to be significant nodes in GSH metabolism. Overall, toxic PAs could be identified by GSTA1 and GPX1 targets pattern, which suggests their common hepatotoxicity mechanism: the interfering of detoxication in GSH metabolism. In addition, all the strategies developed here could be extended to studies on toxicity mechanism of other toxins.

## 1. Introduction

Pyrrolizidine Alkaloids (PAs) are well known as a type of the most hepatotoxic toxins in nature. Currently, more than 660 kinds of isolated PAs and their nitrogen oxide derivatives have been identified in over 6000 plants [1]. PAs mainly appear in four plant families (Boraginaceae, Asteraceae, Leguminosae and Orchidaceae [2,3,4]), and more than half of PAs produce toxicity [3]. Because of their abundance in nature and potent toxicity, including serious hepatotoxicity, carcinogenicity, pneumotoxicity, neurotoxicity and embrotoxicityc [5,6], PAs have received extensive attention.

Decades of efforts have been made to study the hepatotoxicity mechanism of PAs. *In vitro* experiment was regarded as an important approach in studying metabolic toxicity, and cell models including Primary cultured hepatocytes [7,8], L-02 liver cells [9,10], Human hepatoma cell HepG2 [11,12] and Human hepatoma cells HuH-7 [13,14] have been established. In the light of studies on the existing models, it is generally considered that PAs exhibit hepatotoxicity via their activated metabolite, pyrrolic ester, which was catalyzed by the hepatic cytochrome P450 (CYP450) enzyme system [15,16].

The PAs induced hepatotoxicity has long been considered to associate with many factors, such as reactive oxygen species (ROS), CYPs and GSH-metabolizing enzymes [17]. Most recently, accumulating evidences have demonstrated that GSH metabolism plays an important role in regulating the hepatotoxicity induced by PAs. *In vivo* experiments in our team reported that intracellular GSH effect on regulating the hepatotoxicity induced by Adon, Sene and Mone [18,19,20]. In addition, cellular GSH was showed with the ability to prevent hepatotoxicity induced by clivorine [18]. Some studies suggested that oxidative stress plays a crucial role in PAs induced hepatotoxicity [21]. Moreover, it has been showed that the activities of GSH-related antioxidant enzymes were changed by PAs. For instance, isoline induced the change of glutathione *S*-transferase (GST), glutathione peroxidase (GPX) and glycine *N*-methyltransferase (GNMT) [22]. As components of the intracellular GSH system, the activities of GPX, GST and glutathione reductase (GR) decreased significantly on clivorine-treated primary cultured rat hepatocytes [23]. Nine differentially expressed proteins were identified in injured liver of isoline-treated mice. Furthermore, two potential protein targets (GST-pi and ACADM) of isoline were identified as possible signaling molecules related to isoline-induced liver injury [24]. Besides, decreases of GR, GPX and GST activities were induced by monocrotaline [25,26].

However, due to the limitation of current experimental approaches, there are still many PAs of which hepatotoxicity mechanism remains unclear under GSH antioxidant system. If the relationship within the PAs and enzymes involved in the GSH metabolism can be determined systematically, it will lay a solid foundation for future studies concerning PAs hepatotoxicity, and promote the development of related drugs.

In this study, we aim to investigate the common hepatotoxicity mechanism of a large scale of PAs from the three major PAs types in GSH metabolism. Inverse docking and multiple stepwise linear regression were used in mass screening of the potential protein targets of 22 PAs from three major PA types in GSH metabolism. We expect to find the boundary which can distinguish toxic PAs and non-toxic compounds, and consequently uncover the relationship between PAs and liver enzymes in the GSH metabolism. Compared with nontoxic compounds, toxic PAs can be identified by a potential targets pattern, named as GSTA1 and GPX1. It is a possible molecular pattern which can accurately identify the toxic PAs. The molecular mechanisms of the interactions within these two targets and PAs were detected respectively, and the importance of them in GSH metabolism was further confirmed in this study. GSTA1 and GPX1 targets pattern indicates there is a common hepatotoxicity mechanism for toxic PAs, that these toxic PAs could break the balance of free radicals in human body and decrease the excretion of the exogenous toxins by simultaneously interfering two important detoxication pathways related to GSTA1 and GPX1, thus indirectly causing the toxicity on hepatocytes.

## 2. Results

### 2.1. Identifying the Potential Targets of PAs

**Figure 1:**
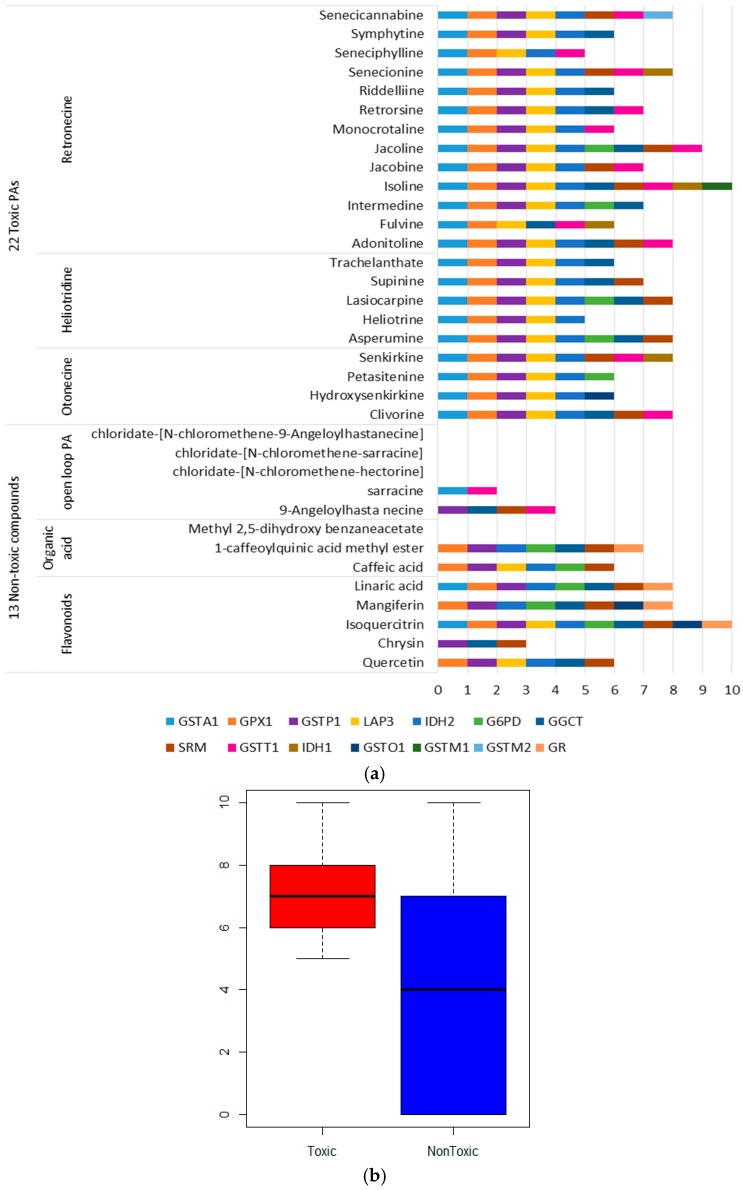
Potential protein targets of Pyrrolizidine Alkaloids (PAs): (**a**) Targets sets of toxic PAs and non-toxic compounds; (**b**) The distribution of the targets number.

In order to identify the potential targets of PAs, two datasets of the small molecules were used to detect the putative targets by reverse docking (Table S1). The positive dataset consists of 22 toxic PAs from three PAs types. For each PA, information on the prototype and its hydrolysis products are included. The negative dataset is composed of five non-toxic open loop PAs and 8 non-PA compounds. These eight non-PA compounds are flavonoids with the same weight to PAs and were extracted as by-products in the extraction of PAs in the laboratory. There are 51 proteases involved in the glutathione metabolism in KEGG [27], 34 of which have 3D structures (Table S2) [28]. Therefore, 35 small molecules were reversely docked to 34 proteases, and 14 proteases were hit. The results were illustrated in Figure 1.

The targets number distribution of the toxic PAs and non-toxic compounds are different (Figure 1b). The potential targets of non-toxic compounds are decentralized, and the median of their targets number is far lower than these of toxic PAs. In contrast, targets of toxic PAs are concentrated, and focused on GSTA1, GPX1, glutathione *S*-transferase pi 1(GSTP1), leucine amiopeptidase (LAP3) and isocitrate dehydrogenase [NADP] (IDH2).

### 2.2. Targets Enrichment of Toxic PAs

To find some commonalities of targets to distinguish toxic PAs from non-toxic compounds, specific targets of toxic PAs were enriched with four stepwise multiple linear regression models. There are four targets enriched in the best model (Table 1), according to the standardized coefficients, GSTA1 and GPX1 weight 50.2% and 69.7% of the toxic prediction precision respectively, while GR and LAP3 weight 68.3% and 33.4% of the non-toxic prediction precision respectively. These targets were combined into different patterns to distinguish the toxic and non-toxic compounds. GSTA1 and GPX1 pattern reached the highest precision with 94.29% (Table 2), indicating toxic PAs’ preference for binding to GSTA1 and GPX1.

### 2.3. Molecular Interactions within PAs and GSTA1, GPX1

**Figure 2:**
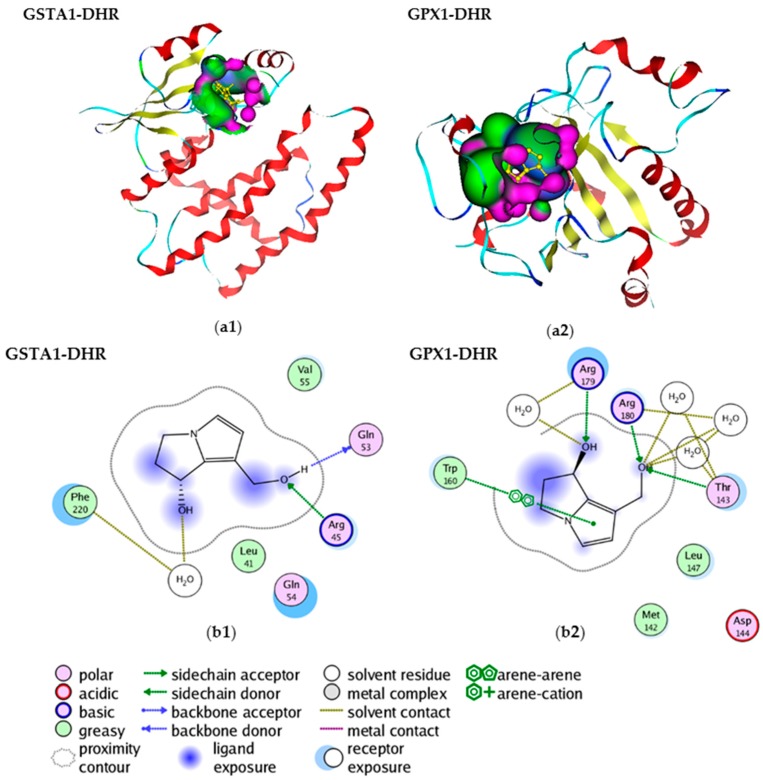
Molecular interactions between dehydroretronecine (DHR) and glutathione *S*-transferase A1 (GSTA1), glutathione peroxidase 1 (GPX1). (**a1**) Docking demonstration of DHR and GSTA1; (**a2**) Docking demonstration of DHR and GPX1. Red ribbon: helix; blue ribbon: loop; yellow ribbon: beta-strand; pink and green: surface; (**b1**) Interaction between DHR and GSTA1; (**b2**) Interaction between DHR and GPX1.

To better understand the interaction of PAs and GSTA1 and GPX1 pattern, active necine bases dehydroretronecine (DHR) (*R*-6,7-dihydro-7-hydroxy-1-hydroxymethyl-5*H*-pyrrolizine) were used to dock GSTA1 and GPX1 respectively (Figure 2a1,a2). The molecular interactions were shown in Figure 2b1,b2. GSTA1-DHR interaction shows that the O on 1-hydroxymethyl of DHR accepts a hydrogen from the Arg45, while the H on the 1-hydroxymethyl donates a hydrogen to Gln53 (Figure 2b1), and OH7 bridges the hydrogen bond with Phe220 side chain via a water molecule. GPX1-DHR interaction shows that the OH on 1-hydroxymethyl accepts two hydrogens from the Arg180 and Thr143, and it also bridges hydrogen bonds with them via water molecules. The OH7 not only accepts hydrogen from Arg179, it also bridges a hydrogen with Arg179 by a water molecule. There also exists a π-π stacking interaction between pyrrole and Trp160 (Figure 2b2). These indicate the molecular mechanisms underlying how toxic PAs interact with GSTA1 and GPX1, and consequently interfere with the activities and functions of them.

### 2.4. Importance of the GSTA1 and GPX1 in GSH Metabolism

**Figure 3:**
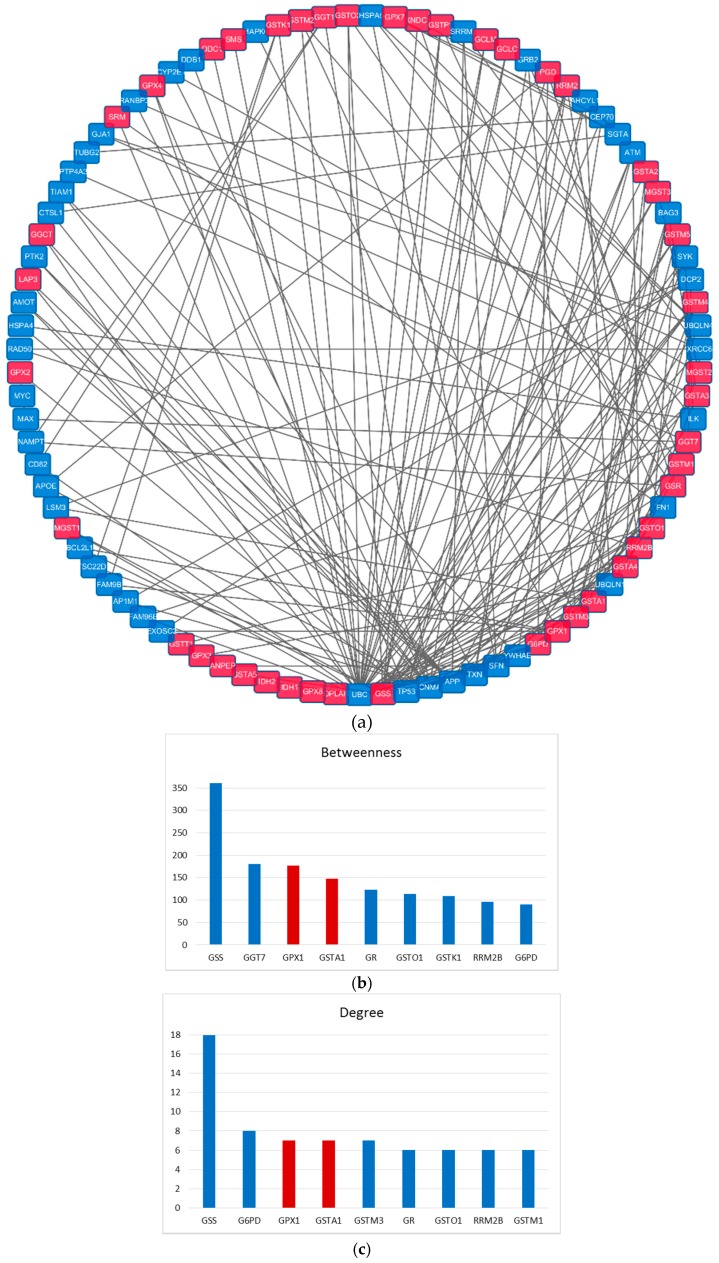

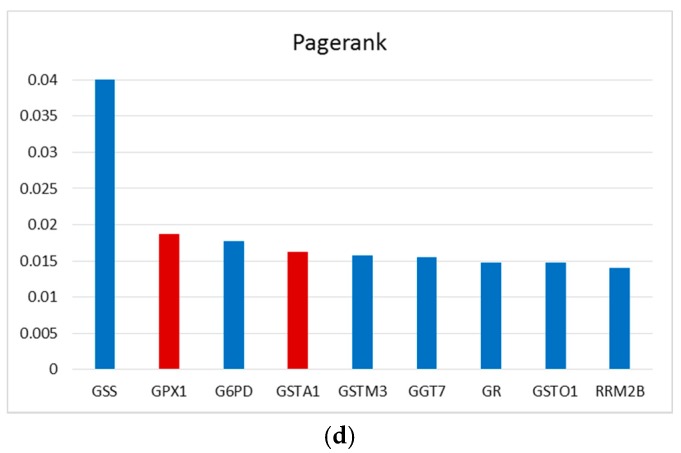
Network analysis of GSTA1 and GPX1 targets pattern. (**a**) protein-protein interaction (PPI) network constructed by 44 enzymes in GSH metabolism; (**b**–**d**) measurements of proteins in network, blue represents the proteases in GSH metabolism; red represents GSTA1 and GPX1.

GSTA1 and GPX1 were analyzed in the protein-protein interaction (PPI) network to confirm their importance in GSH metabolism. A PPI network with 91 proteins and 194 interactions was produced by linking each pair of 44 proteases in GSH metabolism with the shortest path, and the importance of these proteins was measured by betweenness, degree and pagerank (Figure 3a). Under these three measurements, it’s interesting to find that GSTA1 and GPX1 were also the top ranking ones among the 44 proteins in GSH pathway (Figure 3b–d). This suggests that GSTA1 and GPX1 are the crucial nodes in the network, if they were interfered, the interaction networks would be heavily perturbed, which would lead to dysfunction of GSH metabolism.

## 3. Discussion

### 3.1. Toxicity Mechanism Analysis of Toxic PAs Based on GSTA1 and GPX1 Pattern

Our study identified the potential targets of PAs in GSH metabolism, and demonstrated the GSTA1 and GPX1 targets pattern can distinguish toxic PAs from non-toxic compounds with the accuracy over 94%. This pattern suggests toxic PAs probably bind to both these two proteins and influence their function. Furthermore, we have detected potential molecular interactions between secondary metabolites of toxic PAs and GSTA1, GPX1 respectively, which helps understand how PAs affect these two important enzymes.

**Figure 4:**
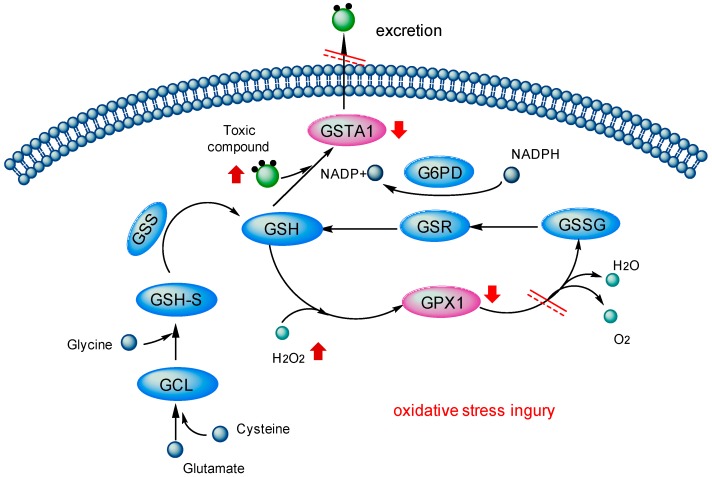
Toxicity mechanism of PAs in glutathione metabolism: downward red arrows stand for the decrease of the activities, upward red arrows for the increase of the activities, black arrows for the direction of biological reactions and red double lines mean this pathway is blocked.

In GSH metabolism (Figure 4), as an important intracellular anti-oxidant, cellular GSH directly takes part in the detoxication of free radicals, carcinogens and peroxides induced oxidative stress [29,30,31,32]. The detoxication effect of GSH could be partly attributed to its role as a cofactor with anti-oxidant enzymes (such as GPX and GST) in accelerating the excretion of toxins [33].

Intracellular GSH could serve as the electron donor in the reduction of peroxides catalyzed by GPX [34]. As a glutathione peroxidase, GPX1 protects cells from oxidative stress by catalyzing the reduction of hydrogen peroxide to water [35,36]. Indirectly, GPX1 is also related to the NADP+/NADPH redox couple, helping adjust the GSH/GSSG ratios to normal level. Generally, GPX1 is considered as a crucial antioxidant enzyme against oxidative stress, and has been found to be effective at removing intracellular peroxides under many physiological conditions [37,38].

Besides, under the catalysis of GST, GSH can conjugate the metabolites of exogenous toxins [39]. GSTs are a class of phase II drug metabolism enzymes, which act on the detoxication of electrophilic compounds capable of generating reactive oxygen species [40,41,42]. They catalyze the conjugation of GSH to these exogenous substrates via a sulfhydryl group-electrophilic center which makes the compounds more water-soluble [43,44]. In this way, they accelerate the excretion of the toxics [45] and decrease the level of oxidative stress, so as to achieve the goal of antioxidant defense and detoxication [46]. GSTA1 has been proved associated with the oxidative stress mediated by hydroxyl radicals (zOH) and the GSTA1 inducers with pro-oxidative potential [47]. It has also been found as a marker of hepatocyte injury in transplantation, toxicity and viral infections [48,49,50].

Taken together, toxic PAs could bind with GSTA1 and GPX1 in liver and interfere two important detoxication pathways in GSH metabolism by decreasing the activities of these two major cellular antioxidant enzymes. This would disrupt the balance of free radicals in the body and hinders the excretion of toxic compounds out of the cell, therefore indirectly resulting in the toxicity on hepatocytes.

### 3.2. Limitations of Reverse Docking

Reverse docking is an important method to calculate and predict the targets of small molecules in a large scale as well as in a non-laboratory environment. However, it yet has two main limitations. On the one hand, it may generate false positives from computing models and docking procedures. On the other hand, there are still no available 3D structures for some receptors, which is the foundation of docking study. Here, only receptors with 3D structures were selected for molecular docking, which may inevitably generate biased results. Besides, there is a need to further optimize computation models and statistical models to decrease false positives. Despite all this, our study could still lay a foundation to future PAs’ toxicity mechanism research, especially to the researches on the detoxication mechanisms regarding the GSTA1 and GPX1.

## 4. Materials and Methods

### 4.1. Dataset

The positive and negative datasets of PAs (Table S1) are retrievable from The Ministry of Education (MOE) Key Laboratory of Standardization of Chinese Medicines, Institute of Chinese Materia Medica, Shanghai University of Traditional Chinese Medicine, Shanghai, China.

The 3D structures of 34 proteins involved in the Glutathione metabolism in KEGG are acquired from protein data bank (PDB) (Table S2).

### 4.2. Identification of Potential Protein Targets of PAs

INVDOCK [51] was used as a reverse docking tool to find the potential targets of PAs. The targets of a small molecule were identified by computer-automatically simulation, by docking the molecule into all cavities of each protein in PDB database (http://www.rcsb.org/) [52,53,54]. Potential targets were selected based on the Affinity (ΔELP) between proteins and ligands according to a specific scoring function. A protein could be considered as a potential target of certain small molecule when ΔELP is simultaneously less than two thresholds [53,55]. The first threshold is the experience score (ΔE Threshold) obtained by statistical analysis of a large number of “protein-ligand” binding energy in PDB database. The other one is the energy score (ΔE Competitor) calculated by scoring function on natural ligand-protein crystal complex.

In this study, only when a protein was identified as the target of all the three states of a same PA, could it be considered as the potential target of this PA. As a result, 14 out of 34 proteins were hit by PAs.

### 4.3. Targets Enrichment of Toxic PAs

Stepwise multiple linear regression analysis was applied to seek the specific targets to distinguish toxic PAs (positive dataset) and non-toxic compounds (negative dataset). Four linear regression models were established based on the targets information, and the differences among them lie in the targets set of positive dataset, shown as follows:

Model 1: Targets set only contains the targets of the PAs prototype.

Model 2: Targets set only contains the targets of DHP.

Model 3: Targets set contains the targets of all small molecules, whether they are PAs prototype, DHP or DHR.

Model 4: Targets set contains the targets which were hit by all the three states of certain PA at the same time.

Key targets filtered out from each of the four models were further combined to predict whether a molecule is a toxic PA. The information of other three models and prediction precisions of different combinations of features are shown in Supplementary Table S3.

### 4.4. Molecular Interactions within Toxic PAs and GSTA1, GPX1

The binding sites and possible molecular interactions within toxic PAs and GSTA1, GPX1 were identified by Simulations module in the commercial software Molecular Operating Environment (MOE).

### 4.5. Importance of the Targets Pattern in GSH Metabolism

In order to investigate the connection of proteases related to the GSH metabolism on the human protein-protein interaction network links (PPI), in particular the importance of GSTA1 and GPx1 pattern, we constructed a subnetwork for GSH metabolism.

Firstly, we constructed a human PPI which composed of 55,363 edges and 10,523 nodes based on 6 PPI databases, including HPRD (Human Protein Reference Database) [56], MINT (the Molecular INTeraction database) [57], BioGRID (Biological General Repository for Interaction Datasets) [58], IntAct (IntAct Molecular Interaction Database) [59], DIP (Database of Interacting Proteins) [60] and MIPS (Mammalian Protein-Protein Interaction Database) [61].

Secondly, we mapped 44 proteases in GSH metabolism on the human PPI and made them interconnected by remaining some background nodes. Under this condition, a GSH metabolism network with 91 nodes and 194 edges was constructed (Figure 3a). Finally, we investigated the importance of the nodes with the measurements of degree, betweenness and pagerank.

## 5. Conclusions

Overall, our study suggests that toxic PAs and their metabolites are most likely to be identified by GSTA1 and GPX1 targets pattern. After binding with GSTA1 and GPX1, toxic PAs could further exert toxicity by breaking the oxidant-antioxidant balance and intercepting detoxication of the exogenous toxins. This pattern could provide some support in the future studies on PAs’ hepatotoxicity mechanism. Moreover, the computational workflow employed in this study may throw light upon the toxic mechanisms studies on other toxins.

## Figures and Tables

**Table 1:** Evaluation of the fourth model.

Coefficients ^a^						
Model	Parameter	Unstandardized Coefficients		Standardized Coefficients	*t*	Sig.
		B	Std. Error	Beta		
1	(Constant)	8.47 × 10^−^^17^	0.100		0.000	1.000
	GSTA1	0.846	0.117	0.777	7.207	0.000
2	(Constant)	0.119	0.079		1.516	0.139
	GSTA1	0.796	0.089	0.731	8.929	0.000
	GR	−0.597	0.115	−0.423	−5.171	0.000
3	(Constant)	−0.067	0.067		−1.005	0.322
	GSTA1	0.471	0.088	0.433	5.340	0.000
	GR	−0.767	0.090	−0.544	−8.526	0.000
	GPX1	0.552	0.101	0.488	5.452	0.000
4	(Constant)	−0.078	0.063		−1.234	0.227
	GSTA1	0.546	0.089	0.502	6.107	0.000
	GR	−0.962	0.121	−0.683	−7.964	0.000
	GPX1	0.858	0.166	0.697	5.183	0.000
	LAP3	−0.364	0.161	−0.334	−2.261	0.031

^a^ Dependent Variable: TOXIC.

**Table 2:** Prediction precision of different targets pattern.

Targets Pattern	Toxic Prediction Precision	Non-Toxic Predictive Precision	Total Prediction Precision
GSTA1	22/22	10/13	91.43%
GPX1	22/22	7/13	82.86%
LAP3	0/22	4/13	11.43%
GR	22/22	5/13	77.14%
LAP3, GR	0/22	2/13	5.71%
GSTA1, GPX1	22/22	11/13	94.29%
GSTA1, GPX1, GR	22/22	0/13	62.86%
GSTA1, GPX1, LAP3, GR	0/22	0/13	0.00%

## Supplementary Materials

Supplementary materials can be found at http://www.mdpi.com/1422-0067/17/3/318/s1.
